# Helminth Eggs as a Magnetic Biomaterial: Introducing a Recognition Probe

**DOI:** 10.3389/fvets.2022.797304

**Published:** 2022-02-23

**Authors:** Ruhollah Shaali, Mohammad Mahdi Doroodmand, Mohmmad Moazeni

**Affiliations:** ^1^Department of Chemistry, Shiraz University, Shiraz, Iran; ^2^Department of Pathobiology, School of Veterinary Medicine, Shiraz University, Shiraz, Iran

**Keywords:** helminth egg, magnetic susceptibility, inductor, water, oxygen

## Abstract

Parasitic helminths, despite their known negative impact (biomaterial) on human health and animal production, have fascinating features. In this study, we find fantastic magnetic properties in several forms: inductor [between 20.10 and 58.85 (±2.50) H], source of detectable electrical voltage [from +0.5 to 7.3 (±0.1) V, vs. the ground, GND, measured by an AVO meter] and different inductor magnitude [between 3.33 and 41.23 (±0.76)] μH, detected by electrochemical impedance spectroscopy as well as frequency scannable electromagnetic wave horn) in several frequencies (including 100, 120, Hz, and 1, 10, 100 kHz) in “Fasciola hepatica”, “*Parascaris equorum*” (with and without larvae), “*Dicrocoelium dendriticum,”* “*Taenia multiceps*”, and “*Moniezia expansa”* eggs. This claim is attributed to some surprising characteristics, including superior inductance and intrinsic magnetic susceptibility. This feature along with a close relationship to helminth egg structure, is a novel probe with acceptable reproducibility (RSD > 8.0%) and high enough trustworthiness for adequate differentiation in their magnitudes, relatively. These traits were measured by the “*Single Cell Rrecording*” methodology using a three-microelectrode system, implanted to each egg at the Giga ohm sealed condition (6.08 ± 0.22 GΩ cm^−1^, *n* = 5). The reliability of these results was further confirmed using multiple calibrated instruments such as a high-resolution inductance analyzer, LCR meter, impedance spectrometer, potentiometer, and an anomalous Hall effect (Magnetic field density) sensor. In addition, the critical role played (Synergistic Effect) by water-like molecules as the intermediate medium, besides the partial influence of other compounds such as dissolved oxygen, are investigated qualitatively, and specific relation between these molecules and magnetic field creation in helminth eggs was proved. These intrinsic characteristics would provide novel facilitators for efficient arriving at the researchable bio-based magnetic biomaterials, besides innovative and real-time identification probes in the “*Parasitology”* fields.

## Introduction

Materials have different responses when an external magnetic field is applied depending on whether they have magnetic properties in their structures (1). If these materials possess magnetic properties, they exhibit specific values for the magnetization (*M*) (2); although to describe the magnetic properties of the material the term “Magnetic Permeability” (μ) is used, which is an acceptable description of how a material behaves concerning an external field (2). Attendance of magnetic moment in the structure of these materials, due to specific atoms, molecules, ions, or domains with a particular spin rotation, has made them more and more attractive in various fields of knowledge (3). According to the bulk magnetic susceptibility or flux density ratio between the interior and exterior of any materials, they can be categorized into five types as follows: diamagnetic (4), paramagnetic (5), ferromagnetic (6), ferrimagnetic (7), and antiferromagnetic (1).

In recent years, magnetic materials have been adopted in different fields of science and technology, such as information storage (8), catalysis (9), magnetic imaging (10), drug delivery (11), separation (12), anti-corrosion (13), and so on (14). In addition, abundant fabrication and research is carried out by scientists on the different types of magnetic sensors including fluxgate-sensors (15), Hall-effect magnetic sensing devices (16), magneto-optical (17), giant magneto-resistive (*GMR*) modules (18), resonance magnetometers (19), and superconducting quantum interference device (*SQUID*) gradiometers (20).

However, there is one essential common point with all these sensors, which is the existence of magnetic materials that operate as their heart. So, synthesis, exploration, and modeling of magnetic materials have great importance because of their spread of uses in the world of technology (21). Nevertheless, significant limitations, tiny magnetic properties, stability, and reproducibility as well as hysteresis of most of the currently existing materials [21], have encouraged scientists; deeper research the magnetic properties of molecules and biological materials such as protein (22), DNA (23), water (24), hemoglobin (25), and magnetotactic bacteria is underway (26). Though, to the best of our knowledge, most of the mentioned figures of merits have not been wholly solved (22–26). It seems that, focusing on biomaterials in the form of inspiration from nature and current existents such as parasites, that, up to now, have only had their negative features evaluated and investigated (27).

The term “*Parasite,”* which refers to the “*Protozoans*,” “*Arthropods*,” and “*Helminths,”* which inhabits the body of another larger animal, the host (27, 28). The contamination of a host by helminth, which can include humans, animals, plants, soils, and even water sources, could cause serious concerns and dangers such as disease, infections, cancers, and even death (29, 30). Despite the negative influence of helminth on their hosts, they are also utilized for the treatment of some diseases such as asthma, atopic eczema, inflammatory bowel disease, multiple sclerosis (MS), rheumatoid arthritis, and type 1 diabetes as well as reducing allergies (31, 32). Serious attention to helminth eggs sometimes shows the survival of them, even in tough conditions such as deficiency of oxygen, nutrient media, light, etc. (33). They are often inactive in these tough conditions (34, 35), nevertheless, the inactive metabolism of these parasites reveals their sophisticated cell physiology (36). At this moment in this research, for the first time, the magnetic field density (*susceptibility*) of helminth eggs are evaluated in detail and this property is attributed to the water and the oxygen molecules present in their structure as the proposed special reagents. These unexpected features, despite viewing surprising attitude(s) in the magnetic technology, are considered as selective probes for the detection, speciation, and recognition of helminth eggs. These characteristics are also comparable with current detection probes such as enzyme-linked immunosorbent assay, etc. (37–39). The magnetic properties of helminth eggs, in addition to introducing them as a new magnetic biomaterial, can be used to distinguish/differentiate them from each other and to inspire scientists to make a new generation of magnetic materials. To the best of our knowledge, up to now, there have not been any introduced materials which completely share the outlined magnetic characteristics.

## Experimental

All required reagents and solutions are reported entirely in the Supplementary Material (see Section Reagents and Materials). All parts of the magnetic parameters of each tested egg are determined based on the best practical designs, which have fully been described in the Supplementary Material (see Section Instruments). This section completely covers all types of methods and instruments used. These designs were based on the “*Single Cell Recording”* methodology (40), at the “*Giga ohm sealed condition*” (41) using “*Implanted Microelectrodes*” (42), whose procedures were comprehensively explained in the Supplementary Material (see Section Procedure).

### Single-Cell Recording Methodology

The magnetic behavior of each helminth egg was evaluated by using a three-microelectrode system including working, pseudo reference, and counter microelectrodes, with the X-Y positions of each egg being set using the X- and Y-axes of the 3-D printer. The microelectrodes were implanted onto the helminth egg shells with 0.0124 ± 0.0008 mm inter-electrode distance (see part Surface area- helminth eggs inter-microelectrode distance), this system was connected to the Z-axis with a triangular shape and using three independent stepper motors (Full cash) and mechanical interfaces, linearly connected to each electrode system, independently. DC/AC voltammetry was applied to the three-microelectrode system, along with potentiostat magnetic parameters measurement on the surface of the working microelectrode vs. the counter one. In order to avoid any unscientific and inaccurate claims about how to acquisition data from different areas of the helminth eggs, the “single-cell recording” methodology has been used.

### Collection of Helminths Eggs

The *Moniezia expansa* eggs were obtained from the gravid proglottids of adult worms collected from the small intestine of naturally infected sheep slaughtered at the “*Shiraz Slaughterhouse*”. *Taenia multiceps* eggs were obtained from the gravid proglottids of adult worms collected from a naturally infected dog referred to the small animal clinic of the School of Veterinary Medicine, Shiraz University (Shiraz, Iran). The *Parascaris equorum* eggs were obtained from specimens referred from the animal clinic at the School of Veterinary Medicine, Shiraz University, Shiraz, Iran. In addition, *Fasciola hepatica* and *Dicrocoelium dendriticum* eggs were collected from the adult worms, obtained from the livers of naturally infected sheep slaughtered at Shiraz slaughterhouse (Zarghan, Fars, Iran).

The uterus of adult female *Parascaris equorum* and uterine area of adult *Fasciola hepatica* and *Dicrocoelium dendriticum* and gravid proglottids of *Taenia multiceps* and *Moniezia expansa* were separately crushed by a mortar and pestle, dissolved in unchlorinated water, and passed through a 500-μm mesh sieve to separate coarse tissue residues from the eggs. Subsequently, the passed materials containing the eggs were washed several times with distilled water. After the total removal of the supernatant, the sedimented eggs were transferred into 2.0-mL microtubes, containing phosphate buffer solution [PBS, (1X)] and stored at 4.0 ± 0.5°C until use. It should be noted that the eggs of Fasciola become embryonated in the environment. However, embryonation takes place only at suitable temperature conditions (e.g., more than 10°C) and humidity. In this study, we collected the Fasciola eggs directly from the uterine area of adult worms and stored them at 4°C until use. Hence embryonation did not occur in the used parasite eggs.

## Results and Discussion

To give a general overview of the helminth eggs, the electrochemical impedance spectroscopy (*EIS*) of each helminth egg was studied via using an electrochemical electron analyzer as a programmable electrical wave (function) generator and data acquisition (sampling) system by applying a single sinusoidal wave with electrical frequency, ranging between 0.1 Hz and 1.0 (±0.1) MHz. For scan (sweep) the electrical potentials, the frequency range divided into 50 sequential parts with logarithmic mode, as the selected frequency step and the electrical wave amplitude as large as 25.00 ± 0.01 (*n* = 3) mV (vs. the total applied potential).

**Figure 1:**
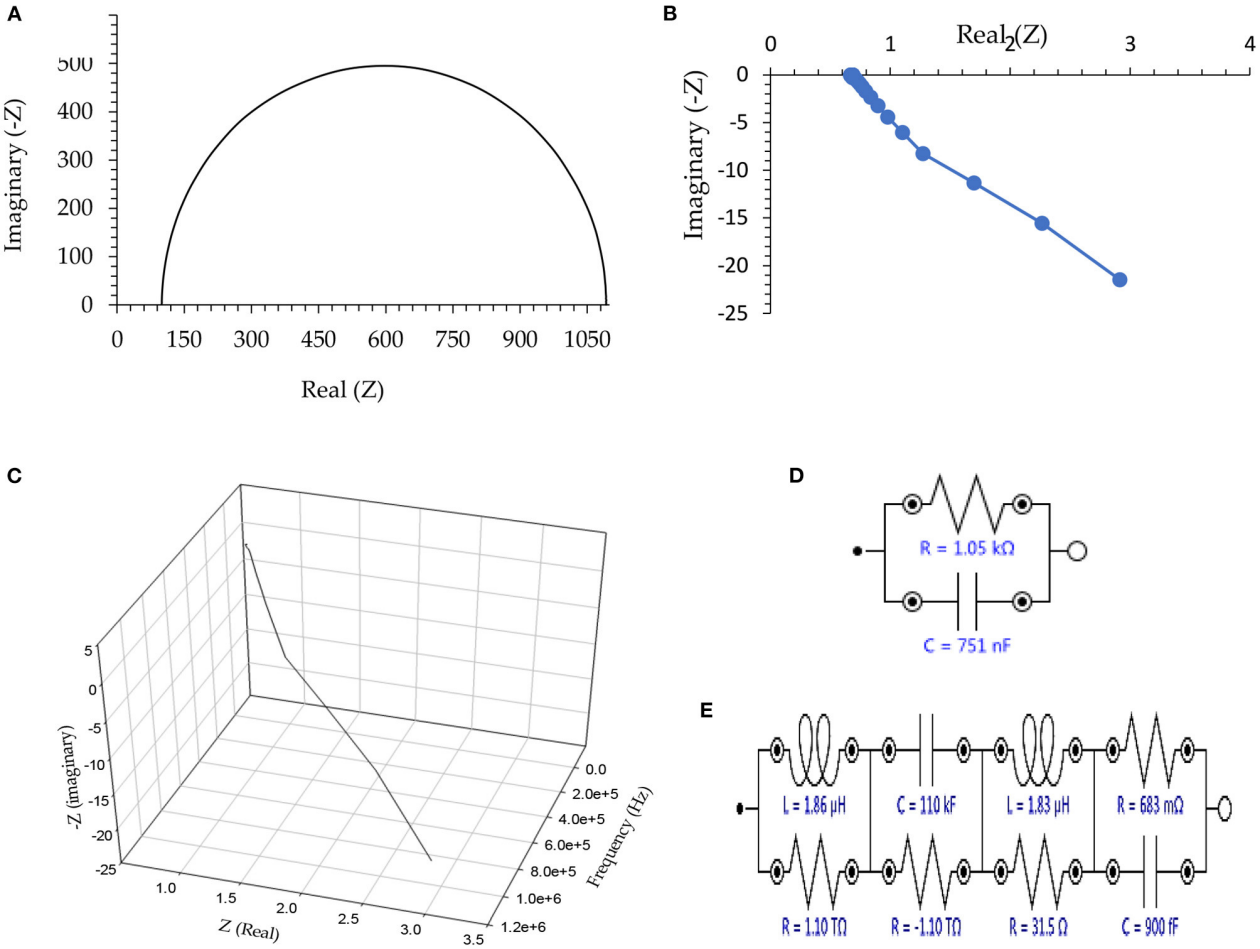
**(A)** Nyquist plot in the absence of any connection to the helminth egg, **(B)** Two-dimensional Nyquist plot for Taenia multiceps, **(C)** Three-dimensional Nyquist plot of Taenia multiceps (as a sample), **(D)** Equivalent circuit as an example for the absence of the helminth egg, and **(E)** Equivalent circuit as an example for Taenia multiceps. Conditions: All conditions and procedures are expressed in detail as mentioned- above during implanting two microelectrodes with 0.0124 ± 0.0008 mm inter-electrode distance, error bar: ±: Standard deviation (*n* = 5).

The Nyquist plots as well as the equivalent circuit, are shown in Figure 1. As shown, elements including electrical capacitance, resistance, inductance, and negative resistance are approved for all the tested helminth eggs. To better display the data, only the output of the experiment on the eggs of Taenia multiceps is presented in Figure 1, however, there is no significant difference between any of the other helminth eggs. However, it should be noted that to simplify results, only the inductor is wholly studied. About these descriptions, it is necessary to note that notations: “1”, “2”, “3”, “4”, “5”, as well as “6” refer to the types of helminth eggs, including “Fasciola hepatica,” “*Parascaris equorum*” (in the absence or presence of any larvae), “*Dicrocoelium dendriticum*,” “*Moniezia expansa*” and “*Taenia multiceps*,” respectively.

According to Figure 1A, in the absence of any electrical connection to helminth egg(s), the EIS showed only the characteristics of the external clamper dummy cell [adopted a regulating (buffer) electronic circuit], which was similar to a regular (*RC*) Nyquist plots (Figure 1D) (43). On the other hand, after setting the single-cell recording connection at the Giga ohm sealed condition, the imaginary part of the Nyquist plot (Figure 1B) showed negative electrical values. This phenomenon, therefore was revealed to have very low capacitance impedance (*X*_*c*_), due to a substantial capacitor (*C*), based on the *X*_*c*_ = *1/(–j.C*.ω*), (*ω = *2*πυ*, j* = $\sqrt{-1}$*)*, formula (44). These results strongly showed a huge inductance impedance (*X*_*L*_), because of a high inductor (*L*) as *X*_*L*_ = *j.L*.ω *(j* = $\sqrt{-1}$) formula (44), and consequently magnetic permeability (μ) (44).

To avoid the complexity in the magnetic analysis of the helminth egg, the existence (role) of different electrical elements in the equivalent circuit of EIS was ignored. Therefore, in the following parts of this report, the only magnetic feature of the helminth egg was evaluated in detail.

### Magnetic Behaviors of the Helminth Eggs

The magnetic inductance of each helminth egg was measured by three distinct procedures:

(i) Directly using a Hall-effect sensing process as a direct low-power magnetic sensing system, briefly *via* estimation of the output potential (Lorentz force) of the clamper dummy cell system (45). This system uses a reference coil, which was directly surrounding each tested helminth egg using a cylindrical magnetic coil as the electromagnetic wave horn. This coil is therefore triggering with an external magnetic field with different electromagnetic flux, which is generated by a magnetic field horn as a pulse/continuous magnetic field generator. The system is stimulated by an initial reference sinusoid with AC sweeping potential (+5.00 ± 0.01 V, vs. GND) at a fixed frequency (250 kHz). The degree of the stimulation depends on the sensitivity, time constant, and accuracy of the Hall effect sensor (46). This is for accessing the reliable measuring the magnetic inductance values of each helminth egg based on the magnetic flux gradient using the Lenz rule (47) (Related formula: At the initial coil: ΔB_Initial_ = –L × (δi/δt), ΔB_egg_ α ΔB_Initial_, δi_egg_/δt = –ΔB_egg_/L_egg_, ΔV_out_ = δi_egg_ × R_Halleffectsensor_) (48).
(ii) Inductance analysis of each helminth egg, utilizing the LCR meter.
(iii) Inductance element measurement via following the degree of the inductor element the related equivalent circuit using EIS by sweeping the AC frequency from 0.1 to 100.0 KHz.

**Figure 2:**
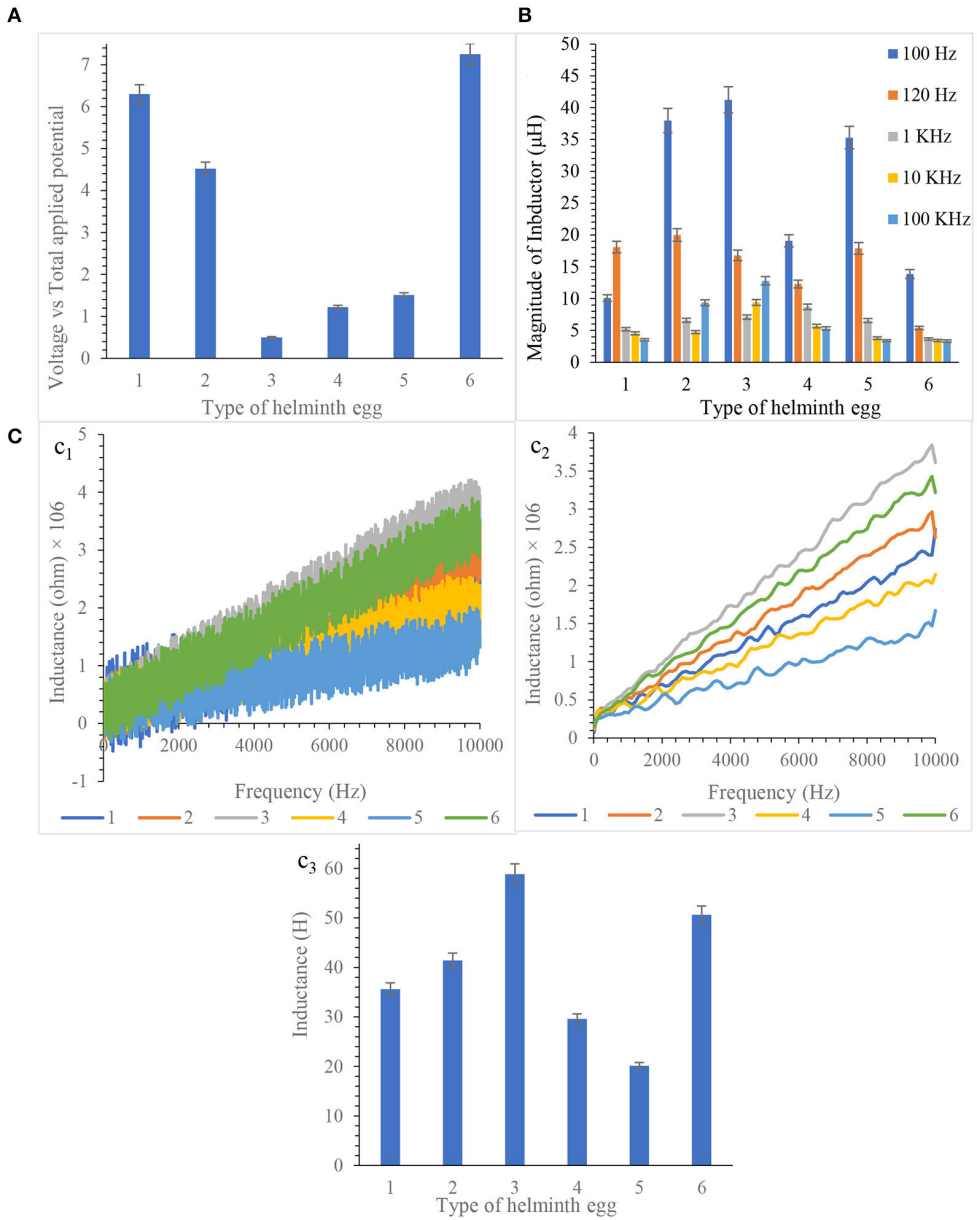
**(A)** Hall effect measurement at 250 kHz frequency, **(B)** Inductance determination by LCR meter, and **(C)** Inductance measurement by frequency scanning from 10.0 MHz to 10.0 Hz based equivalent circuit (c_1_ Non- smoothed, c_2_ smoothed, c_3_ Final Results). Note that, to prevent clutter, error bars are eliminated. Measurements taken during five repetitions implanting three microelectrodes with 0.0124 ± 0.0008 mm inter-electrode distance, error bar: ±: Standard deviation (*n* = 5).

By exhaustively checking and analyzing the results, it is concluded that each helminth egg processed significant magnetic properties. All results are illustrated in Figure 2.

### Semi-Qualitative Interpretation

According to scientific studies reported so far, biological compounds such as proteins (49), lipids (50), phenolic compounds (51), carbohydrates (52), water (53), and oxygen (54) are often present in the structure of helminth eggs. However, accurate claims, arrangement, and spatial orientation of these biological units in the structure of helminth eggs have not still been wholly reported (to the bests of our knowledge). Nevertheless, only some studies briefly lookingat the helminth egg formation have been reported (55, 56).

To understand the role of the materials of the helminth egg in the abovementioned parameters, helminth eggs were precisely weighed once without and once with heating up to 60.0 ± 1.0°C inside the vacuum oven at 720.0 ± 0.3 Torr pressure along a 5.0 h time interval. According to Table 1, the weight ratio between the water removed and the remaining material was an exact match. After applying the heating procedure, all the tests were again repeated to find out the effect(s) of the water-molecules removed materials. As clearly illustrated in Table 1 (first column on the right), all the reported parameters have experimentally been approached to zero (inferior) values, consequently, each egg had acted as a non-repeatable electrical resistor.

**Table 1:** Effect of lost weight on the electrical parameter.

**Type of helminth egg**	**Fresh average weight ±SD (*n* = 10) (g)**	**Dried average weight ±SD (*n* = 10) (g)**	**Removed average weight/fresh average weight ±SD (*n* = 10)**	**Inductor quantity after drying (H) ±SD (*n* = 10)**
Fasciola hepatica	0.0034 ± 0.0028	0.0018 ± 0.0015	0.44 ± 0.00	1.47 ± 0.82
*Parascaris equorum* without larvae	0.0054 ± 0.0027	0.0049 ± 0.0024	0.09 ± 0.00	1.13 ± 0.76
*Parascaris equorum* with larvae	0.0038 ± 0.0025	0.0027 ± 0.0018	0.28 ± 0.00	1.04 ± 0.84
*Dicrocoelium dendriticum*	0.0031 ± 0.0023	0.0014 ± 0.0004	0.52 ± 0.00	1.16 ± 0.57
*Moniezia expansa*	0.0058 ± 0.0011	0.0024 ± 0.0004	0.53 ± 0.00	1.92 ±0.83
*Taenia multiceps*	0.0054 ± 0.0029	0.0023 ± 0.012	0.56 ± 0.00	1.34 ± 0.51

*During five repetitions for weighting and during implanting three microelectrodes with 0.0124 ± 0.0008 mm inter-electrode distance, error bar: ±: Standard deviation*.

The materials released from the system, especially water, play a significant role during the creation of magnetic behavior. Under the above reports, the removed material mostly contained water medium, oxygen, and sometimes carbon dioxide from their metabolism. Although water molecules don't show any magnetic behavior due to their diamagnetic property, according to research and the presence of water and lipids layer in the helminth egg structure (57); it can therefore be deduced the magnetic behavior of helminth eggs arises from the creation of a thin layer of water next to the thin layer of lipid molecules.

However, the oxygen in the structure of the helminth eggs (58, 59) also has attribution in the magnitude of a magnetic field, due to its high magnetic susceptibility (60). Therefore, the magnetic field originated from this component (60, 61) as well as water layers (61). So, according to this special behavior, the helminth eggs showed significant magnetic properties. It seems that the general magnetic property of the helminth eggs are based on the presence of dissolved oxygen in the structure of water adsorbed to the vitelline cells as well as the layer of oriented water molecules near the lipid layer. It appears orientation and configuration of different tissues, especially proteins, made 3-D distributed vitelline cells, which are considered as an independent magnetic domain, in various series and parallel modes (62).

To further evaluate the influential roles of the water medium on the magnetic performance of the eggs, two procedures were applied to each tested egg. The first one was based on the suspension (soaking) of a previously dried egg (according to the recommended procedure) in a water medium (100.0 mL) at different temperatures, ranging between 25 and 50°C, in both dark and light conditions.

The second was related to the presence of some ionic species, such as aqueous NaCl solution with a 0.10 mol L^−1^ concentrations for at least 10.0 h. Assitionally, the second procedure was performed *via* situating the helminth eggs inside a closed bottle (glass balloon, 50.0 mL) containing water aerosol with relative humidity (*RH*) higher than 60 %, generated using a humidifier system over a long time (>10.0 h). However, the lack of any observable magnetic behaviors on the helminth egg event after applying at least one drying procedure to each tested egg pointed to the influential role(s) of the water medium as well as the significant (and irreversible) influence(s) of desorbed water on the denaturation (*Physiogentic*) of these eggs. In addition to the heating mentioned above, ethanol was also used to inactivate the parasite eggs, the experiments were repeated, with the result being the same when heating.

## Conclusions

Much research has been performed on bio-magnetic materials to date; this study presents a bio-magnetic structure that is available for use in a variety of scientific and technological fields. In this study, a suitable method for detecting the parasite eggs without any intervention of the operator is expressed. In further addition, the critical role of water molecules and dissolved oxygen (as the special reagents) in the creation of a magnetic field have been semi-qualitatively proved. In conclusion, the above reported intrinsic characteristic of the helminth eggs would provide novel facilitators for efficient arrival at researchable bio-based magnetic elements, besides innovative and real-time identification probes in the “*Parasitology”* fields in the near future.

## Data Availability Statement

The original contributions presented in the study are included in the article/Supplementary Material, further inquiries can be directed to the corresponding author/s.

## Author Contributions

MD directed the research group and supported the necessary methods and edited the manuscript. RS consulted the project, analyzed the data, edited the manuscript, performed all the electrical experiments, analyzed the data, and wrote the manuscript. MM conceptualized the study, collected the helminth eggs, and edited the manuscript. All authors contributed to the article and approved the submitted version.

## Funding

This study was financially supported by Shiraz University.

## Conflict of Interest

The authors declare that the research was conducted in the absence of any commercial or financial relationships that could be construed as a potential conflict of interest.

## Publisher's Note

All claims expressed in this article are solely those of the authors and do not necessarily represent those of their affiliated organizations, or those of the publisher, the editors and the reviewers. Any product that may be evaluated in this article, or claim that may be made by its manufacturer, is not guaranteed or endorsed by the publisher.
