# Supplementary material for: Helminth Eggs as a Magnetic Biomaterial: Introducing a Recognition Probe
Source: Front Vet Sci. 2022 Feb 23;9:797304. doi: 10.3389/fvets.2022.797304 (PMC8904871; doi:10.3389/fvets.2022.797304)
Supplement: Supplementary file 1 [file Data_Sheet_1.docx]

***Supplementary Information***

**Related to**

**Helminth eggs as huge magnetic biomaterial: Introducing a recognition probe**

Ruhollah Shaali^1^, Mohammad Mahdi Doroodmand^1,*^, Mohmmad Moazeni^2^

^1^ Department of Chemistry, Shiraz University, Shiraz, Iran

^2^ Physiological division of Department of basic science, School of Veterinary Medicine, Shiraz University, Shiraz, Iran

*Correspondence: [doroodmand@shirazu.ac.ir](mailto:doroodmand@shirazu.ac.ir), doroodmand@yahoo.com (M. M. Doroodmand), Tel: +98-71-36137152, Fax: +98-71-36460788

**Reagents and Materials**

All the reagents were purchased in their analytical grades. The solution of 1.00 ± 0.01 % (W/V) of NaCl (99.9 %, Merck Company), this solution was prepared by deionized water (DIW or de-ionized water, resistivity: of 17.5 ± 0.2 million ohm-cm, Direct-Q® Water Purification System, France). Ultra-analytical grade of ethanol (C_2_H_5_OH, 99.0 %, Merck Company) was used to kill (destroy) the helminths eggs.

**Instruments**

The adopted instrumentation systems included as mentioned in Table 1.SP:

**Table 1.SP |** Instruments and devices.

| A cubic faraday’s cage: (Dimension: 50.0 × 50.0 × 60.0 cm) |
| --- |
| Electroanalyzer (Autolab, ECI10M, frequency range: 10.0 MHz to 100.0 (±0.1) µHz Input impedance: 100.0 GΩ, Metrohm AG, Ionenstrasse, CH-9100 Herisau, Switzerland) |
| Hall effect sensor (Hall Effect Sensor Chip A0201f Dip-4 E-era Electronics) |
| Autoclave (NanBei, Qingdao, China) |
| Stainless Steel Microelectrode (Epoxy-insulated stainless-steel microelectrode, type: 308, diameter: 0.010 in., length: 3 in., tapered tip size: 12 °, A-M Systems^TM^, 131 Business Park Loop Sequim, WA 98382, U.S.A., Catalog #: 572700) |
| Inverted fluorescence microscope (Trinocular head; Top video port, Germany) |
| **Table 1.SP\| Cont’d** |
| Glue paste (Vistamaxx Performance Polymers, High Elastic TPU Hot Melt Adhesive Film Thermo, 1.75 ± 0.05 mm, Shenzhen Tunsing Plastic Products Co., Ltd., China) |
| 3-dimensional printer (Design via modification of the three-dimensional printer, Pxmalion, B07DYN48FZ, PX-MINI, Accuracy: (X, Y, and Z dimension): 0.01 mm, Position accuracy: 0.01 mm, dimension: 30×30×20 cm, resolution: ± 0.01 mm, LulzBot TAZ 6, USA) |
| Oscilloscope (DS-5422 (200MHz, 2GS/s, 2CH, Max. 500k-points Digital Storage Oscilloscope, DS5400-5600 Series, Iwatsu, English) |
| Dummy cell (Autolab, RC circuit, R: 1 KΩ, C: 1 µF, Metrohm^AG^ Company) |
| Function generator (GW Instek GFG-3015 Dual Display Programmable Function Generator with External Counter, 15MHz Frequency, GFG-3015, USA), Programmable Potentiostat/Galvanostat function generator (SRS DS345, China) |
| Cylindrical coil: (Height: 5.0 cm, radius: 2.0 cm, 12.0 V, AC, 4.0 A, Military Series, Analog Device, UK), Reference coil (Ring coil, dimension: 5 × 5 cm, Charging Coil, Wireless, AWCCA-50N50 Series, 6.3 ± 0.1 µH, ± 2%) |
| Digital Micrometer (Mitutoyo 395-353 - MIC, DIG SPH FACE, 3"/76.2MM, Spherical Face Micrometer, Series 395 Digital model with a spherical anvil, US) |
| Glue film (2×2 cm, Zhejiang Shichuang Optics Film Manufacturing Co., Ltd., China), Glue film (Vistamaxx Performance Polymers, High Elastic TPU Hot Melt Adhesive Film Thermo, 1.75 ± 0.05 mm, Shenzhen Tunsing Plastic Products Co., Ltd., China) |
| High-speed Charge Coupled Device, (CCD camera system (Full HD, 1000X (Manual), USB Adjustable Digital Microscope Camera Magnifier with 8 LED, light-emitting diode, illuminator, Picture Resolution: 1960×1080, Video Capture Resolution: 0.3-2.0MP, Frame Rate: 30 f/s under 600 LUX Brightness, Model Number: S1000 X, Guangdong, China), (Elmo 9711X3 model TNC4614X High-Resolution CCD camera Color, Samsung, Assembled in Japan)) |
| **Table 1.SP\| Cont’d** |
| Reference voltage generator: (ADR510, SOT-23-3 package, Analog Devices, Fluke 752 A, PO Box 9090, Everett, WA 98206 USA), Reference voltage generator: (US4788455A–CMOS, programmer function, generator, Analog Device, US) |
| Mechanical Interface (Piezoelectric Micrometer Screw, P-853 • P-854, high-resolution travel: 25 µm, PI-Japan) |
| LCR meter (GW Instek, 900 Series Handheld USA) |
| Micro stepper motor (Size: 42.3 mm square × 48 mm, CHANGZHOU JKONGMOTOR CO., LTD., China) |
| Coaxial wires (RG6, CIMPLE CO - 30' Feet, USA), |
| BNC, connector, China) |
| Potentiometer (Multi-turn potentiometer: 100.00 KΩ, military Series, China) |
| Low noise shunt-mode reference input potential (Accuracy: ±0.35%, ultralow noise: 4.0 µV_p-p_ (vs. total applied potential), temperature drift: 70 ppm/°C) |
| Ultra-high resolution gravimeter (Analytical Balance, AE 240, Mettler, USA) |

Note: The instruments were calibrated based on their related protocol, prior each analysis.

**Helminth definition:**

***Moniezia expansa:*** The adult worms may reach 600 cm in length and 16 mm in width. The scolex is mostly 0.36-0.80 mm wide having prominent suckers. A mature segment contains two sets of male and female sexual organs, ovary and vitelline glands form a ring. Inter proglottidal glands across the width of segments. Gravid proglottid contains a sac like uterus filled with a large number of fertilized eggs. The eggs are somewhat triangular, measuring 56-67 µm in diameter.

***Taenia multiceps:*** Adult worms are 40-100 cm long. In mature segments, poral lobe of ovary is not smaller than aporal lobe. In gravid segments, the uterus has 14 to 20 lateral branches filled with eggs. Each gravid segment may contain 37,000 eggs. The eggs are 29 - 37 µm in diameter and containing a hexacanth embryo (oncosphere) when laid.

***Parascaris equorum:*** It is a stout, large, thick (up to 8 mm) and yellow-white nematode. Adult females may reach up to 50 cm in length. The parasites' eggs are subglobular, 90-100 µm in diameter, brown in color with a thick sticky and albuminous covering.

***Fasciola hepatica:*** Adult worm is leaf-shaped, greyish brown in color and 2–3.5 cm long with a width of up to 13 mm. It has a large cephalic cone at the anterior end having a small oral sucker at the end of cephalic cone. The eggs are broadly ellipsoidal, operculated and measure 130–150 µm long by 60–90 µm wide.

***Dicrocoelium dendriticum:*** With 8–14 mm length and 1.5–3 mm width, the adult worm has a lanceolate shape. The eggs are small (35–45 × 20–30 µm), ovoid, dark brown in color, operculate and embryonated when laid.

**Technique**

High-frequency impedance spectroscopy included Autolab system (ECI10M, frequency range: 10.0 MHz to 100.0 (±0.1) µHz, ***Input impedance***: 100.0 ± 0.1 GΩ, Metrohm AG, Ionenstrasse, CH-9100 Herisau, Switzerland).

**Procedure**

In order to design an experiment for determination of magnetic property of helminth eggs, by inductometer, LCR meter, and the *EIS*, the below setup was adopted. Briefly, the adhesive glue film was connected to the two horizontal directions (i.e. X and Y axes) plane of the three-dimensional printer. After that, the selected helminth egg was immobilized and fixed on the surface of the glue film (as shown in Figure S1). The direct electrical connection to the support as the electrode system was also set via controlling the size of the adhesive glue. After proper drying time for (1.0 h per each mL of the fluid), the analysis process was operated. The Giga ohm sealed condition was then provided according to the suggested procedure. After that, the magnetic parameters were measured by the recommended procedures. The optimization is based on the one-at-a-time method.


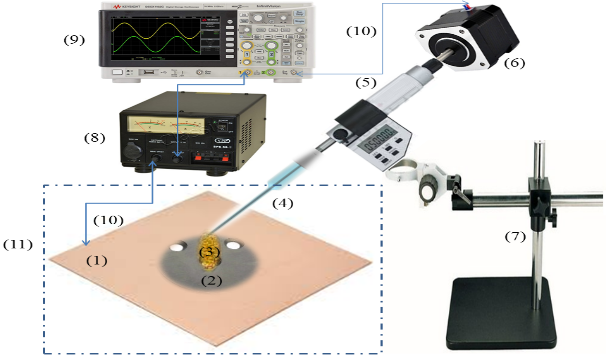


5 cm

**Figure S1. |** Schematic of the dummy cell designed to estimate the magnetic feature of the helminth egg. 1) copper plate, 2) sample holder well (diameter: 2.00 mm, height: 1.50 mm), 3) helminth egg, 4) microelectrode, 5) mechanical interface (digital micrometer), 6) stepper motor and driver, 7) base, 8) AC switching power supply, equipped with capacitive AC voltage divider circuit dummy cell, 9) oscilloscope, 10) coaxial wires, and 11) faraday’s cage. The analysis was achieved at standard temperature and pressure (*STP*) conditions.

**Helminth egg’s Giga ohm sealed condition**

The Giga ohm sealed condition (G*Ω*/cm) of each helminth’s egg was provided prior to the single cell recording via procedure below:

Direct measurement using a resistance tester connected the working and counter microelectrode system with 0.0124 ± 0.0008 mm inter-electrode system as the ohmmeter probes [1]

**Ohmmeter at zero current condition for Giga ohm sealed conditions estimation**

In order to achieve the Giga ohm sealed condition according to the reported procedure [2], the electrical resistance between the implanted microelectrodes on the egg’s shell measured after their direct forward and backward movement via using a stepper motor, by applying a resistometer. Under these conditions and in the presence of a Faraday’s cage, the following ohmic value was obtained (6.18 ± 0.12 GΩ/cm (n=5)).

**Micro-electrode implant**

Microelectrode implantation were performed in the Z-cantilevered arm of the 3-D printer with the inter-electrode distance of 0.0124 ± 0.0008 mm by directly connection of microelectrodes to the helminth egg based on the single cell recording method. This process led to detecting the microtip position using the CCD camera as well as the simultaneous measuring of the helminth egg cell electrical resistance (Ω cm^-1^) to reach the Giga ohm sealed condition.

**Part: *Surface area***

**Helminth eggs inter-microelectrode distance**

In order to achieve inter-microelectrode distance was estimated based on the HR-AFM imaging (Figure S2a). According to the voltage profile imaging (Figure S2b), the average inter-electrode distance was estimated to be 0.0124 ± 0.0008 mm (During 5 sequential estimations).


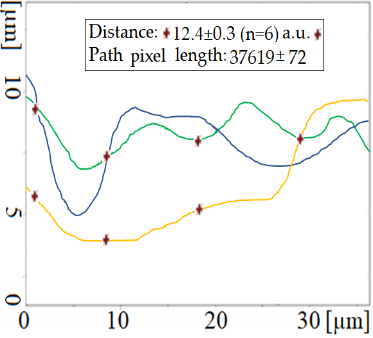

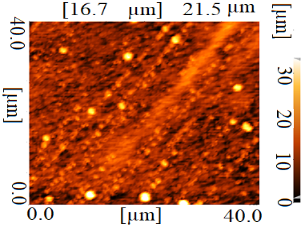


a

b

**Figure S2.** | a) HR-AFM, b) voltage profile image of Fasciola hepatica egg during estimation of the inter-microelectrode distance. **Condition:** The analysis was achieved on the AC mode on the alumina support.

**References:**

[1] P. Molnar, J.J. Hickman, Patch-clamp methods and protocols, Springer Science & Business Media2007.

[2] M. Malboubi, Y. Gu, K. Jiang, Study of the tip surface morphology of glass micropipettes and its effects on giga-seal formation, Electronic Engineering and Computing Technology, Springer2010, pp. 609-619.
